# Molecular profiling of satellite cell heterogeneity during differentiation in an individual turkey pectoralis major muscle

**DOI:** 10.1016/j.psj.2025.105735

**Published:** 2025-08-25

**Authors:** Joseph Yimiletey, Zhenyang Li, Chunmei Wan, Sandra Velleman, Hui Yu

**Affiliations:** Department of Animal Sciences, The Ohio State University, Columbus, OH, United States

**Keywords:** Satellite cell, Heterogeneity, Turkey pectoralis major muscle, RNA-sequencing, Differentiation

## Abstract

Satellite cells are muscle stem cells that contribute to post-hatch muscle growth by fusing with adjacent muscle fibers, thereby promoting muscle fiber hypertrophy. Satellite cells are not a uniform population; even within a single muscle, such as the turkey *pectoralis major*, they exhibit substantial variability in their proliferative capacity. To better understand this cellular heterogeneity, we performed transcriptomic profiling of individual satellite cell clones isolated from the same turkey *pectoralis major* muscle after 48 hours of differentiation. These clones had been previously categorized as early (fast-growing) or late (slow-growing) based on their proliferation rates. Principal Component Analysis revealed a clear separation in transcriptomic profiles between early and late clones, with over 1,400 genes differentially expressed. Gene ontology analysis indicated that early clones were enriched for genes involved in organ development and receptor signaling, while late clones upregulated genes associated with cell proliferation and peptidase activator activity. Notably, among the top 10 most significantly differentially expressed genes, *MSTN* was highly upregulated in early clones, while *FGFBP1* showed elevated expression in late clones. *MSTN* is a well-established negative regulator of satellite cell proliferation and differentiation, whereas *FGFBP1* modulates FGF signaling, thereby promoting both proliferation and differentiation. Both genes play critical roles in regulating satellite cell differentiation, and their differential expression patterns have important implications for breast muscle growth. Despite these differences, expression levels of *PAX7* and other key myogenic regulatory factors remained comparable between the two groups, suggesting that their core myogenic identity was preserved. Collectively, these findings underscore the intrinsic genetic heterogeneity of satellite cell populations within a single muscle during differentiation. Together with our previous results showing marked transcriptional differences after 72 hours of proliferation, these data provide consistent evidence that turkey satellite cells maintain distinct molecular identities across both proliferative and differentiative stages.

## Introduction

Meeting the nutritional demands of a growing population requires advancements in genetic improvement strategies to increase overall meat production. According to the U.S. Census Bureau, the U.S. population has grown by nearly 58 million people, with an average annual growth rate of approximately 0.8 % (Wilder, 2024). The poultry industry, which produces the most widely consumed meat in the United States, continues to experience increasing demand. Over the past decade, per capita consumption of poultry products has risen steadily and is projected to continue growing. Specifically, per capita turkey consumption nearly doubled from 8.1 pounds in 1970 to 14.8 pounds in 2023 (NASS, 2023). This rising demand has driven breeders and producers to focus on maximizing growth efficiency, particularly in lean carcass development, especially the breast muscle (**p. major)**, which is the most economically valuable part of the carcass. According to the United States Department of Agriculture (NASS, 2023), the total turkey production reached approximately 218 million birds in 2023, contributing an estimated $6.57 billion to the poultry industry. These stats highlight the critical role of genetic advancements in improving production efficiency and ensuring the poultry industry can effectively meet increasing consumer demand.

In poultry, the mechanisms governing skeletal muscle development differ between embryonic and post-hatch stages (Hartley et al., 1992; Liu, et al., 2020). During embryonic development, muscle growth primarily occurs through the proliferation and differentiation of myoblasts, which fuse to form primary muscle fibers (Bentzinger, et al., 2012; Halevy, et al., 2000). The total number of muscle fibers is largely established before hatching. After hatching, muscle growth transitions from hyperplasia (an increase in fiber number) to hypertrophy (an increase in fiber size) (Halevy, et al., 2004). This hypertrophic growth primarily depends on satellite cells (**SCs**, muscle stem cells) proliferation, differentiation and fusion with existing muscle fibers by donating their nuclei to increase protein synthesis potential (Moss and Leblond, 1970). The first week post-hatch is a critical period characterized by maximal mitotic activity of SCs (Mozdziak, et al., 1994). During this time, nutritional challenges and thermal stress can significantly affect the biology of SCs, negatively influencing muscle growth and resulting in reduced muscle mass accumulation and impaired meat quality (Velleman, 2023).

Satellite cells, located between the basal lamina and sarcolemma of a muscle fiber, were first identified by Mauro (1961). They respond immediately to muscle injury and serve as the primary mediators of muscle repair and regeneration (Tidball, 2005). Moreover, SCs are not a homogeneous population of cells. For instance, Khodabukus, (2015) discovered that SCs originating from slow and fast myofibers retain the contractile protein expression and metabolic profiles of their source fibers. During muscle regeneration, SCs undergo asymmetric division, producing two distinct daughter cells: one preserving its ability for self-renewal while the other dedicated to proliferation and differentiation to support muscle repair (Kuang, et al., 2007; Ono, et al., 2010). Additionally, SCs obtained from turkey lines with different growth rates exhibit distinct transcriptomic profiles and varied responses to thermal stress (Reed, et al., 2022a, 2022b). Notably, SCs isolated from growth-selected turkey lines demonstrate greater hypertrophic potential, and significantly larger myotube diameters (Clark, et al., 2017; Velleman, et al., 2003; Velleman and Coy, 2020; Xu, et al., 2021). The turkey p. major muscle is composed exclusively of Type IIb fibers (Miller, et al., 1985; Wiskus K.J., 1976; Yost, et al., 2002) . However, McFarland et al. (1995) demonstrated notable diversity among SCs within this uniform muscle type. In their study, a total of 73 individual SCs were isolated from the p. major muscle of a 6-week-old Nicholas tom turkey. These SCs exhibited significant variations in growth rates, with the fastest-growing cells reaching 65 % confluency in 17 days (early clones), whereas the slowest-growing cells required up to 30 days to achieve the same level (late clones). Further analyses revealed that the rapidly proliferating SC populations exhibited increased expression of fibroblast growth factor receptors (*FGFR)*, suggesting enhanced responsiveness to fibroblast growth factors (Yu, et al., 2025). These cells also produced more heparan sulfate proteoglycans during differentiation (McFarland, et al., 2003). Additionally, a follow-up study found that the early clones exhibited greater sensitivity to the inhibitory effects of transforming growth factor beta on both proliferation and differentiation compared to the late clones (Yun, et al., 1997).

Based on these previous findings, we hypothesized that the early and late clones isolated from the same p. major muscle represent two genetically distinct populations with inherently different transcriptomic profiles. These genetic differences are likely responsible for distinct response to biological stimuli, impacting crucial processes such as proliferation, differentiation, and sensitivity to growth factors and signaling molecules. In a recent study, we compared the transcriptomic difference between early and late clones after 72 hours (**hrs**) proliferation (Yu, et al., 2025). Our results identified over 5,300 differentially expressed genes between the two cell clones. Gene ontology analysis revealed that genes highly expressed in early clones are essential for muscle development and structural maturation, while those upregulated in late clones play roles in cell-cell communication, extracellular matrix interactions, and cytokine activity - key factors for maintaining a functional satellite cell niche. These findings highlight the genetic and functional diversity of SCs in turkey p. major muscle. In the current study, we aimed to determine whether the genetic and functional diversity observed among these two SC clones during proliferation persists during differentiation. Therefore, we analyzed the transcriptional differences between the two SC clones following 48 hours of differentiation. The key molecular pathways and mechanisms that distinguish the two SC clones were characterized using gene functional analysis. Gaining insight into SC-mediated processes driving p. major muscle growth will aid in developing strategies to enhance animal growth and meat production.

## Materials and methods

### Turkey myogenic satellite cells

The satellite cell clones used in this study were previously established by McFarland et al. (1995). Satellite cells were isolated from the p. major muscle of a 6-week-old Nicholas tom turkey and cultured in McCoy’s 5A cell growth medium. Individual cells were picked and transferred to a 96-well culture plate using the Quixell cell manipulator robotic system (Stoelting Co., Wood Dale, IL). This system employs a micropipette to selectively transfer single suspended SCs into wells for clonal expansion. Clones were categorized based on their proliferation rates: those reaching confluency within 17–19 days in a 25 cm² tissue culture flask were considered as early clones, while those requiring 28–29 days were classified as slow clones, reflecting a slower growth rate. All clones were preserved in liquid nitrogen for future research.

The early and late clones were cultured following the procedures outlined in (Reed, et al., 2017b). Briefly, equal numbers of cells were plated, and incubated at 38°C in a plating medium containing Dulbecco’s Modified Eagle’s Medium (D5523, Sigma Aldrich, St. Louis, MO), 10 % chicken serum (C5405, Sigma Aldrich, St. Louis, MO), 5 % horse serum (H1270, Sigma Aldrich, St. Louis, MO), 1 % antibiotics-antimycotics (30004CI, Corning, New York, NY), and 0.1 % gentamicin (GT-10, Omega Scientific, Tarzana, CA). After 24 hours of attachment, the cells were cultured in McCoy’s 5A growth medium (M9309, Sigma-Aldrich, St. Louis, MO) supplemented with 5 % horse serum, 5 % chicken serum, 1 % antibiotic-antimycotic solution, and 0.1 % gentamicin. After 72 h of proliferation, the growth medium was replaced with a DMEM differentiation medium containing 3 % horse serum, 1 % antibiotics-antimycotics, 0.1 % gentamicin, 0.1 % gelatin, and 1 mg/mL bovine serum albumin (BSA, Sigma-Aldrich). After 48 hours of differentiation, the medium was removed, cells were washed twice with phosphate-buffered saline (PBS), and plates were stored at −80°C for subsequent RNA extraction using TRIzol^TM^ Reagent (15596018, ThermoFisher, Waltham, WA).

### RNA isolation and sequencing

Total RNA was extracted using the TRIzol method per the manufacturer’s protocol. The quality and concentration of extracted RNA were assessed at The Genomics Shared Resource at The Ohio State University using the Agilent 2100 Bioanalyzer. Only RNA samples with an RNA Integrity Number (RIN) above 9 were sent to Innomics Inc. (Sunnyvale, CA) for sequencing. Stranded paired-end 150 bp reads were generated using the DNBSEQ platform, with three replicates per cell line.

### RNAseq data analysis

Raw FASTQ sequencing data quality was assessed using FastQC (v0.12.1) (S. FASTQC. ). Reads were aligned to the turkey reference genome (UMD 5.1, ENSEMBL Annotation 111) using the STAR aligner (v2.7.11b) under default settings (Dobin, et al., 2013). Quality control assessments were conducted using QualiMap (v2.3) (Okonechnikov et al., 2016), confirming high-quality alignment across all samples. Differential gene expression analysis between early and late clones was performed using DESeq2 (Love, et al., 2014).

Gene annotation was performed following a multi-source approach. Primary gene identifiers were obtained from the ENSEMBL database. Where ENSEMBL IDs were missing, additional annotations were sourced from the research of Dr. Kent M. Reed and his team (Reed, et al., 2017a, 2022a, 2022b). Gene ontology (GO) analysis was conducted using g: Profiler (Kolberg et al., 2023), accessible at https://biit.cs.ut.ee/gprofiler/gost. Only genes exhibiting a log fold change (logFC) greater than 1 and a *P*-value below 0.05 were considered significant. The reference genome for analysis was Meleagris gallopavo.

### Quantitative PCR analysis

Total RNA was extracted using TRIzol Reagent according to the manufacturer’s instructions. First-strand cDNA synthesis was then performed using 1 μg of total RNA with random primers and M-MLV Reverse Transcriptase (Invitrogen, 28025013). Quantitative real-time PCR (qRT-PCR) was conducted on all samples using the QuantStudio3 PCR system (Thermo Fisher Scientific) and SYBR Green Master Mix (Applied Biosystems). Primer sequences are listed in Supplementary Table 1, and all primers were used at a final concentration of 250 nM. Relative gene expression was analyzed using the 2^−ΔΔCT^ method.

### Statistical analysis

Data are presented as means ± standard error of the mean (SEM), with sample sizes specified in the figure legends and main text. Statistical analyses were performed using GraphPad Prism 9.0 software (GraphPad Software, San Diego, CA, USA). For comparisons between two groups, an unpaired two-tailed Student’s *t*-test was used. Specific statistical methods applied to each analysis are described in the corresponding figure legends. A *P*-value of < 0.05 was considered statistically significant.

## Results

### Summary of overall gene expression

Total RNA was extracted from early clones (*n* = 3) and late clones (*n* = 3), with each sample consisting of pooled material from three wells to generate individual barcoded libraries. Sequencing yielded over 191 million 150 bp paired-end reads (SRA BioProject: PRJNA1280141). Read counts per library ranged from 28.1 to 35.9 million, with an average of 31.8 million reads (Table 1). On average, more than 98.7 % of nucleotides had a quality score above Q20, and 96.5 % exceeded Q30. Replicate libraries showed high consistency across samples.

**Table 1:** Summary of RNA-seq data from the 48-hour differentiation experiment For each library, the total number of raw reads, Q20 and Q30 quality scores (%), the number of detected genes (defined as genes with >1 mapped read), and the percentage of uniquely mapped reads are reported.

Cell Line	Replicates	PE reads	Q20(%)	Q30(%)	GC Content (%)	Observed Genes	Uniquely mapped reads %
Early Clones	1	31,790,486	98.73	96.50	48.44	14,083	81.61 %
	2	28,176,952	98.63	96.21	48.50	14,033	82.73 %
	3	31,148,392	98.80	96.71	48.54	14,143	82.31 %
Late Clones	1	32,152,022	98.71	96.46	48.78	14,023	81.95 %
	2	35,994,785	98.74	96.54	48.82	14,123	84.30 %
	3	31,822,575	98.78	96.65	48.44	14,024	85.13 %

**Fig. 1:**
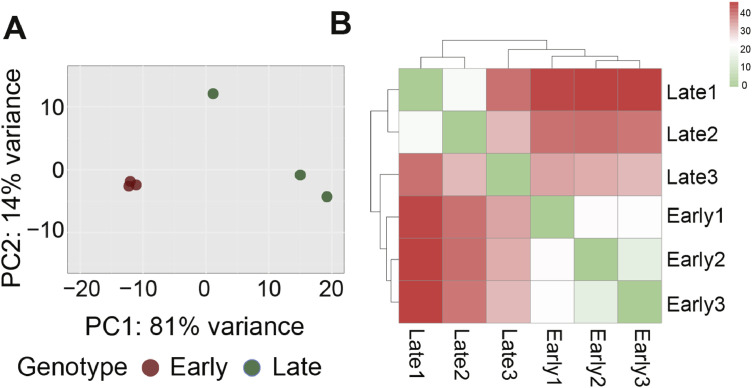
**(A)** Principal component analysis (PCA) plot showing separation of early and late cell clones based on the first (PC1) and second (PC2) principal components of gene expression. **(B)** Heatmap of the sample-to-sample distance matrix illustrating clear dissimilarity between early and late clones and high similarity among replicates within each clone type.

On average, 14,072 genes showed evidence of expression (defined as at least one mapped read per library), with comparable numbers detected in early clones (14,086) and late clones (14,057). More than 83 % of reads were uniquely mapped to the turkey genome (Table 1). Principal component analysis (PCA) based on normalized read counts revealed clear separation between early and late clones along the first two principal components (Fig. 1A), indicating substantial transcriptional differences between the two cell populations. Technical replicates clustered tightly as nearest neighbors in the PCA space, validating the pooling strategy for expression analysis. Heatmap clustering further demonstrated a clear distinction between early and late clones while maintaining consistent intra-group relationships (Fig. 1B), reinforcing the observed transcriptomic divergence between the two cell types.

### Gene expression analysis

**Fig. 2:**
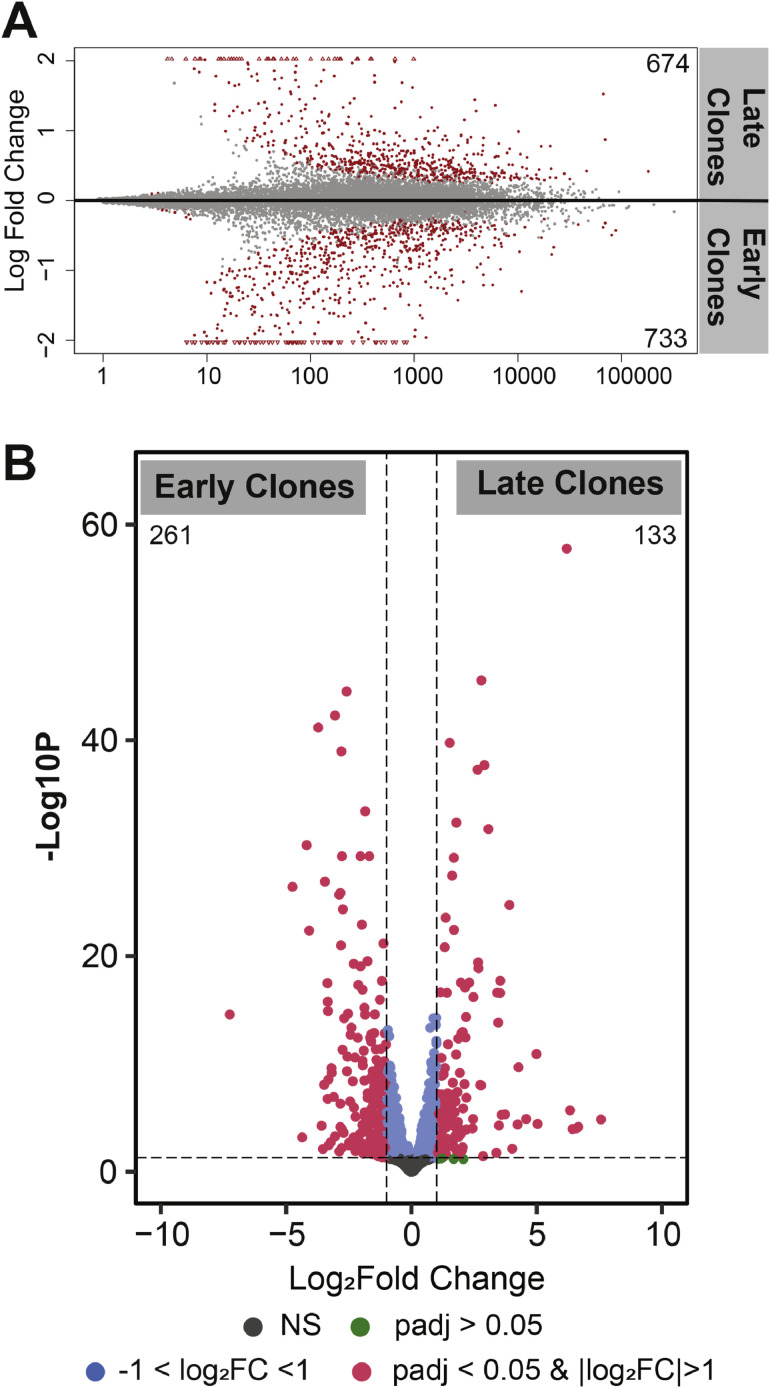
**(A)** MA plot (Bland-Altman plot) illustrating gene expression differences on a log scale. Genes with a *P*-value < 0.05 are highlighted in red, and the total number of upregulated genes is indicated for each clone. **(B)** Volcano plot showing the distribution of differentially expressed genes. Genes meeting the thresholds of *P* < 0.05 and |log_2_FC| > 1 are marked in red.

Differences in gene expression between early and late clones are illustrated by the distribution of uniquely and commonly expressed genes in the Bland-Altman (MA) plot shown in Fig. 2A. This plot displays the log fold change (M) versus the average expression (A) for each gene. A total of 1,407 differentially expressed genes (DEGs) were identified with an adjusted *P*-value < 0.05, including 674 genes upregulated in late clones and 733 upregulated in early clones. Applying a more stringent cutoff (|Log_2_FC| > 1 and adjusted *P* < 0.05) further narrowed this to 133 genes upregulated in late clones and 261 in early clones. These results highlight marked transcriptional differences between the two cell populations, as visualized in Fig. 2B.

### Gene ontology analysis

**Fig. 3:**
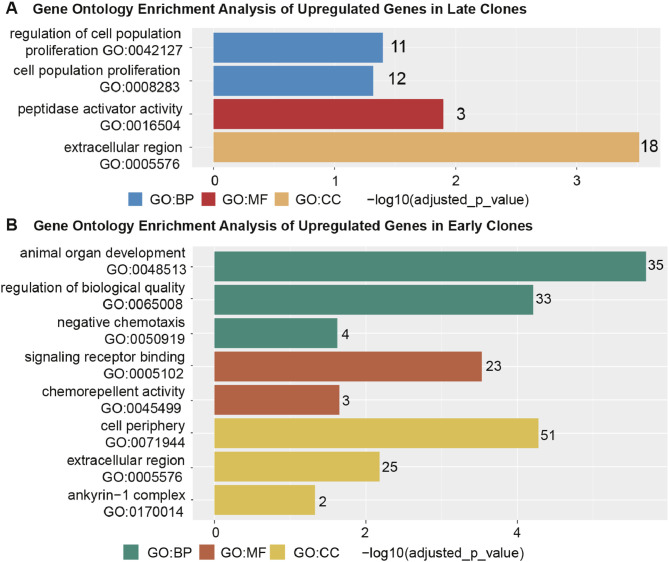
The horizontal bar chart illustrates the top three ontology terms, categorized into CC (Cellular Components), MF (Molecular Functions), and BP (Biological Processes). The x-axis represents the -log_10_ (adjusted *p*-value), while the numbers displayed on each bar indicate the number of genes associated with each term. **(A)** Functional analysis of genes upregulated in late clones. **(B)** Functional analysis of genes upregulated in early clones.

To characterize the functional profiles of genes upregulated in early and late clones, we performed Gene Ontology (GO) enrichment analysis using g: Profiler (g:GOSt Functional Profiling), with *Meleagris gallopavo* (turkey) specified as the input organism. The analysis was conducted across the Biological Process (BP), Molecular Function (MF), and Cellular Component (CC) categories using differentially expressed genes that met the criteria of |log₂FC| > 1 and adjusted *P* < 0.05 (Fig. 3).

In early clones, GO terms related to cell population proliferation and its regulation were enriched (−log_10_ (adjusted *P*-value) = 1.4), indicating increased proliferative activity. Enrichment of peptidase activator activity (−log_10_ (adjusted *P*-value) = 1.9) and the extracellular region (−log_10_ (adjusted *P*-value) = 3.5) suggest involvement in proteolytic processes and extracellular interactions associated with early clone activation. In contrast, late clones exhibited enrichment in distinct GO terms, with upregulated genes associated with animal organ development (−log_10_ adjusted *p*-value = 5.7), regulation of biological quality (−log_10_ adjusted *P*-value = 4.2), and negative chemotaxis (−log_10_ adjusted *P*-value = 1.6), suggesting a shift toward differentiation and tissue organization. Enriched molecular functions included signaling receptor binding (−log_10_ adjusted *P*-value = 3.5) and chemorepellent activity (−log_10_ adjusted *P*-value = 1.6). In the cellular component category, terms such as cell periphery (−log_10_ adjusted *P*-value = 4.2), extracellular region (−log_10_ adjusted *P*-value = 2.2), and ankyrin-1 complex (−log_10_ adjusted *P*-value = 1.3) indicate involvement in membrane-associated structures and signaling pathways.

### Top ten differentially expressed genes between early and late clones

**Fig. 4:**
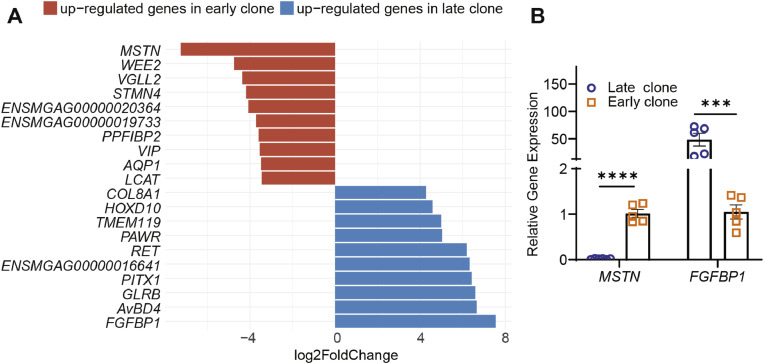
**(A)** Top 10 differentially expressed genes between early and late clones, presented with their Log_2_ fold change values (adjusted *P*-value < 0.05). **(B)** qRT-PCR analysis confirmed the significant upregulation of MSTN in late clones and FGFBP1 in early clones.

The top 10 most significantly differentially expressed genes for each clone type are shown in Fig. 4A. Among the genes upregulated in early clones, several are associated with myogenic differentiation and structural maturation. Notably, *MSTN* (*myostatin*), a member of the transforming growth factor beta (TGF-β) superfamily and a well-established negative regulator of muscle growth(Carnac, et al., 2007; McPherron, et al., 1997), is unexpectedly elevated in early clones (fast-growing cells) - possibly serving as a feedback mechanism to restrain excessive differentiation and proliferating and fine-tune myogenic progression. *VGLL2* (also known as *VITO1* or vestigial-like family member 2) is a protein-coding gene that plays an important role in muscle fiber formation during chicken embryonic development(Hou, et al., 2022; Li, et al., 2023). Its expression has been shown to increase during the differentiation of muscle cells(Li, et al., 2023). Studies using *Vgll2* knockout mice have demonstrated its role in adult skeletal muscle, where deletion of *Vgll2* resulted in an increased proportion of fast-twitch type IIb fibers and a downregulation of slow-twitch type I myosin heavy chain expression(Honda, et al., 2017). *WEE2*, a known cell cycle inhibitor in germ cells of rodents and humans, was also upregulated. Its function in chickens has not yet been reported. In humans, spontaneous mutations in the *WEE2* gene have been associated with total fertilization failure in women(Hanna, et al., 2020). *STMN4* (*stathmin-like 4*) is essential for maintaining the neural progenitor cell pool in the dorsal midbrain(Lin and Lee, 2016; Ritchie, et al., 2025). Although its role in muscle has not been specifically studied, it is involved in microtubule remodeling and may contribute to cytoskeletal reorganization necessary for the morphological changes during myotube formation.

In contrast, genes upregulated in late clones suggest a profile more characteristic of a niche-responsive or less differentiated state. *FGFBP1*, which enhances FGF signaling, supports the maintenance of satellite cell proliferation and inhibits premature differentiation(Gonzalez, et al., 2020). Recent studies suggest that FGFBP1 promotes presynaptic maturation of developing neuromuscular junctions and helps delay their degeneration during aging(Boido and Vercelli, 2016; Taetzsch, et al., 2017). In chickens, *AvBD4* belongs to a family of 14 avian β-defensins-antimicrobial peptides known for their broad-spectrum activity and key role in the innate immune response(Sugiarto and Yu, 2004). The *GLRB* gene encodes the β subunit of the glycine receptor, which plays a key role in inhibitory neurotransmission. Mutations in *GLRB* have been associated with muscle rigidity, tremors, and myoclonic jerks in mice (Grudzinska, et al., 2005; Molon, et al., 2006).

To independently validate the transcriptomic data, quantitative reverse transcription PCR (qRT-PCR) was performed to quantify the expression of *MSTN* and *FGFBP1*, which represented the most significantly differentially expressed genes in early and late clones, respectively. Consistent with the RNA-seq results, qRT-PCR analysis revealed a significant upregulation of *MSTN* in early clones and elevated expression of *FGFBP1* in late clones (Fig. 4B).

### Comparative expression of *PAX7* and myogenic regulatory factors in early and late clones

**Fig. 5:**
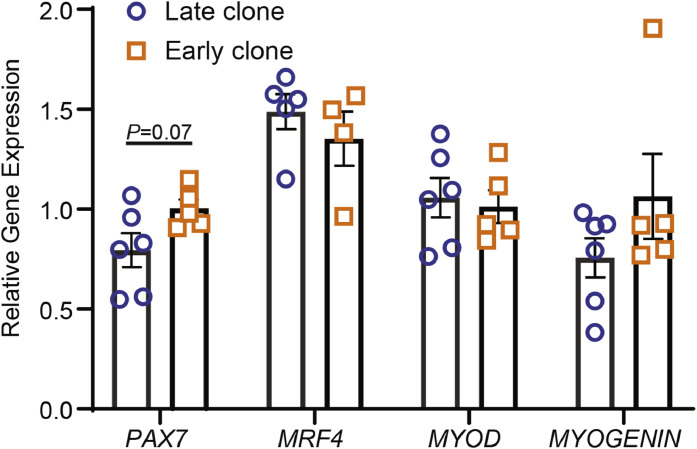
Comparison of PAX7 and myogenic regulatory factor expression between early and late clones.

We examined the expression of *PAX7* and key myogenic regulatory factors (*MYOD, MYOGENIN*, and *MRF4*) to assess the myogenic capacity and differentiation potential of early and late clones, and to rule out whether any differences observed in the RNA-sequencing data could be attributed to the variations in differentiation stages (Fig. 5). *PAX7* is a well-established marker of quiescent and proliferating satellite cells(Halevy, et al., 2004; Seale, et al., 2000), while myogenic regulatory factors (MRFs) are essential transcriptional regulators of myogenic lineage progression(Buckingham and Rigby, 2014; Hernandez-Hernandez, et al., 2017). *MYF5* and *MYOD* function as early-acting MRFs involved in satellite cell activation and proliferation, whereas *MYOGENIN* and *MRF4* are associated with later stages of differentiation and myotube formation(Hawke and Garry, 2001; Rawls, et al., 2000; Smith, et al., 1993). Although not statistically significant (*P* = 0.07), early clones exhibited a trend toward higher *PAX7* expression compared to late clones (Fig. 5). Both cell clones showed comparable expression levels of *MRF4, MYOD*, and *MYOGENIN,* while *MYF5* expression was undetectable under the conditions of this study.

## Discussion

Satellite cells are essential for skeletal growth, repair, and regeneration. Increasing evidence indicates that these cells constitute a heterogeneous population (Tierney and Sacco, 2016), differing in embryonic origin (Gros, et al., 2005), muscle fiber association (Mauro, 1961), developmental stage (Relaix, et al., 2006), surface marker expression (Relaix, et al., 2006), and functional properties such as proliferative capacity and regenerative potential. In the biomedical field, SC heterogeneity has been extensively studied, particularly with the advent of single-cell RNA sequencing, which has enabled the identification of distinct subpopulations with specialized transcriptomes (Dell'Orso, et al., 2019), (Barruet, et al., 2020), and (Sousa-Victor, et al., 2022) However, in the field of poultry research, satellite cell heterogeneity remains poorly characterized. This is largely due to the lack of appropriate research tools, including robust methodologies for identifying novel, poultry-specific satellite cell subtypes, validated antibodies for detecting distinct subpopulations, and transgenic reporter models for *in vivo* characterization. The absence of these resources continues to hinder progress in understanding satellite cell diversity and its implications for muscle growth and development in poultry.

Our study investigated SC clones originally isolated and characterized by McFarland et al. from the p. major muscle of a single turkey. These clones exhibited distinct proliferation rates and have previously been shown to respond differentially to fibroblast growth factor receptor 1 signaling (McFarland, et al., 2003) and to exhibit varying sensitivities to transforming growth factor beta(Yun, et al., 1997). In our recent transcriptomic analysis comparing the same early and late clones after 72 hrs of proliferation(Yu, et al., 2025), we identified substantial differences in gene expression. Specifically, over 5,000 genes were differentially expressed, with 2,675 genes upregulated in late clones and 2,672 genes upregulated in early clones. Gene ontology analysis from two independent sources revealed marked functional divergence: early clones were enriched for pathways related to muscle structure formation and cytoskeletal organization, whereas late clones showed enrichment in pathways associated with extracellular receptor interactions, cell communication, signaling, and cytokine activity. These pronounced differences prompted us to investigate whether such divergence persists during differentiation. Compared to the 72-hour proliferation phase, transcriptomic profiling after 48 hours of differentiation from the current study revealed a reduced number of differentially expressed genes with 674 genes upregulated in late clones and 733 in early clones (Fig. 2). During differentiation, myoblasts exit the cell cycle, fuse, and form multinucleated myotubes(Sampath, et al., 2018). It is expected that cells undergoing differentiation are synchronized through shared regulatory pathways that govern the initiation and maintenance of this process(Hindi, et al., 2013). Such coordination may reduce the transcriptional heterogeneity observed during proliferation. Nevertheless, we still observed over 1,400 genes differentially expressed between the two cell clones, indicating that intrinsic genetic differences persist even during differentiation.

Analysis of the top 10 most significantly differentially expressed genes in each clone revealed a striking upregulation of *MSTN* in early clones (fast-growing cell, Fig. 4A). This result was unexpected, as it is generally anticipated that fast-growing cells would exhibit elevated expression of genes that promote SC proliferation and differentiation. In contrast, *MSTN* is well recognized as a potent negative regulator of SC proliferation and differentiation (Garikipati and Rodgers, 2012; McCroskery, et al., 2003; McFarland, et al., 2006, 2007), and its inhibition is known to promote muscle hypertrophy(Catipovic, 2004; Kim, et al., 2020; Lee, et al., 2020). This result was further validated by qRT-PCR analysis (Fig. 4B). The elevated expression of MSTN in these fast-growing early clones may reflect a regulatory mechanism aimed at restraining excessive proliferation and differentiation. In this context, *MSTN* may act as a fine-tuning modulator, balancing the expansion of progenitor cells with their timely commitment to the myogenic lineage.

Comparing the top 10 most significantly DEGs identified in this study with the top 20 DEGs from the 72-hour proliferation(Yu, et al., 2025), we found that six genes-*AvBD4, RET, GLRB, TMEM119, PAWR*, and *PITX1* were consistently upregulated in late clones across both conditions. Although *FGFBP1* was not among the top 20 DEGs in the previous study, it was significantly elevated in late clones during the 72-hour proliferation (Log_2_FC = 4.45). This overlap on the DEGs suggests that these genes may serve as signature markers of the late clone phenotype, potentially defining intrinsic molecular characteristics that are independent of the satellite cells’ biological state, whether proliferating or differentiating. It may also imply a stable transcriptional program in late clones, possibly indicative of a distinct regulatory or developmental trajectory. In contrast, this level of consistency was not observed in early clones, where the top DEGs varied more substantially between the two conditions. This variability may reflect greater transcriptional plasticity in early clones, with gene expression more dynamically influenced by the cells’ biological context. It is worth noting that we quantified the transcript levels of *MYOD, MYOGENIN*, and *MRF4* in both clones (Fig. 5). The comparable expression of these key myogenic regulators suggests that the two clones were at similar stages of myogenic progression, thereby ruling out asynchronous differentiation as the primary cause of the observed transcriptional differences.

Despite the critical role of satellite cells in muscle development, their biology - particularly their heterogeneity - remains poorly characterized in poultry species, especially turkeys. Recent studies have reported increased muscle fiber damage in the p. major muscle of growth-selected turkeys, which has been associated with significant meat quality issues in the turkey industry. These include conditions resembling pale, soft, and exudative meat in swine, as well as deep pectoral myopathy and focal myopathy(Hocking, 2014; Owens, et al., 2009; Velleman, et al., 2003; Zampiga, et al., 2020). Such abnormalities are likely driven by disruptions in normal muscle development. Advancing our understanding of SC heterogeneity and function in turkeys will be essential for uncovering the cellular mechanisms underlying these myopathies and for developing effective strategies to enhance muscle integrity and meat quality in commercial turkey production.

## Conclusion

In conclusion, our findings demonstrate that early and late SC clones -representing the fast-growing and slow-growing populations, respectively - maintain distinct transcriptional identities even after 48 hrs of differentiation. A previous study characterized marked transcriptional differences during 72 hrs of proliferation. The persistence of these differences suggests that intrinsic molecular programs, rather than external cues alone, play a central role in defining satellite cell heterogeneity. Compared to the 72-hour proliferation phase, the late clone exhibits a more stable and consistent DEG profile across biological states, whereas the early clone displays greater transcriptional plasticity. These insights underscore the importance of satellite cell heterogeneity in the development of the p. major muscle and highlight the need for further investigation into the regulatory mechanisms that govern satellite cell fate and function.

## Data avalibility

The datasets presented in this study can be found in online repositories. The names of the repository/repositories and accession number(s) can be found below: https://www.ncbi.nlm.nih.gov/genbank/, PRJNA1280141.

## Declaration of generative AI and AI-assisted technologies in the writing process

During the preparation of this work the author(s) used Copilot in order to correct any grammar mistakes, polish and improve the clarity, coherence, and overall readability of the scientific paper. After using this tool/service, the author(s) reviewed and edited the content as needed and take(s) full responsibility for the content of the publication.

## CRediT authorship contribution statement

**Joseph Yimiletey:** Writing – original draft, Methodology, Investigation, Formal analysis, Data curation. **Zhenyang Li:** Methodology, Formal analysis, Data curation. **Chunmei Wan:** Resources, Methodology, Investigation. **Sandra Velleman:** Writing – review & editing, Resources, Conceptualization. **Hui Yu:** Writing – review & editing, Writing – original draft, Visualization, Supervision, Methodology, Investigation, Funding acquisition, Formal analysis, Data curation, Conceptualization.

## Disclosures

The authors declare that they have no known competing financial interests or personal relationships that could have appeared to influence the work reported in this paper.

## Appendix Supplementary materials

Image, application 1
