# Supplementary material for: Molecular profiling of satellite cell heterogeneity during differentiation in an individual turkey pectoralis major muscle
Source: Poult Sci. 2025 Aug 25;104(11):105735. doi: 10.1016/j.psj.2025.105735 (PMC12419073; doi:10.1016/j.psj.2025.105735)
Supplement: Supplementary file 1 [file mmc1.docx]

Supplementary Table 1. Primer sequences for real-time quantitative polymerase chain reaction

| Gene | Sequence (5’- ‘3) | Product size | GenBank access number |
| --- | --- | --- | --- |
| *MRF4*  *MYOGENIC REGULATORY FACTOR 4* | AGGCTCTGAAAAGGAGGACTGT  AGGCTGCTGGAAGCCGACGACT | 307BP | NM_001303141.1 |
|  |  |  |  |
|  |  |  |  |
| *PAX7*  *PAIRED BOX 7* | AGGCTGACTTCTCCATCTCTCC  TGTAACTGGTGGTGCTGTAGGTG | 156BP | NM_205065.1 |
|  |  |  |  |
| *MYOD*  *MYOGENIC DIFFERENTIATION* | GATGGCATGATGGAGTACAG  AGCTTCAGCTGGAGGCAGTA | 201BP | NM_001303171.1 |
|  |  |  |  |
| *MSTN*  *MYOSTATIN* | AAACGGTCCCGCAGAGATTT  CAGGTGAGTGTGCGGGTATT | 195BP | NM_001303161.1 |
|  |  |  |  |
| *MYOGENIN* | CCTTTCCCACTCCTCCCAAA  GACCTTGGTCGAAGAGCAACT | 175BP | NM_001303170.1 |
|  |  |  |  |
| *GAPDH GLYCERALDEHYDE-3-PHOSPHATE DEHYDROGENASE* | GAG GGT AGT GAA GGC TGC TG  CCA CAA CAC GGT TGC TGT AT | 200BP | U94327.1 |
|  |  |  |  |
| *FGFBP1 FIBROBLAST GROWTH FACTOR-BINDING PROTEIN 1* | CCATTGATGTTACGTACTCTTTGC  ACATAACTGAGGAGTGCATAGC | 110BP | XM_003205921.4 |
|  |  |  |  |
|  |  |  |  |
